# Medial prefrontal cortex serotonin 1A and 2A receptor binding interacts to predict threat-related amygdala reactivity

**DOI:** 10.1186/2045-5380-1-2

**Published:** 2011-09-27

**Authors:** Patrick M Fisher, Julie C Price, Carolyn C Meltzer, Eydie L Moses-Kolko, Carl Becker, Sarah L Berga, Ahmad R Hariri

**Affiliations:** 1 Center for Neuroscience, University of Pittsburgh, Pittsburgh, Pennsylvania 15260, USA; 2 Center for the Neural Basis of Cognition, University of Pittsburgh, Pittsburgh, Pennsylvania 15260, USA; 3 Department of Radiology, University of Pittsburgh, Pittsburgh, Pennsylvania 15260, USA; 4 Department of Radiology, Emory University, Atlanta, Georgia 30322, USA; 5 Department of Psychiatry, University of Pittsburgh, Pittsburgh, Pennsylvania 15260, USA; 6 Department of Gynecology & Obstetrics, Emory University, Atlanta, Georgia 30322, USA; 7 Department of Neuroscience & Psychology, Duke University, Durham, North Carolina 27710, USA; 8 Institute for Genome Sciences & Policy, Duke University, Durham, North Carolina 27710, USA

## Abstract

**Background:**

The amygdala and medial prefrontal cortex (mPFC) comprise a key corticolimbic circuit that helps shape individual differences in sensitivity to threat and the related risk for psychopathology. Although serotonin (5-HT) is known to be a key modulator of this circuit, the specific receptors mediating this modulation are unclear. The colocalization of 5-HT_1A _and 5-HT_2A _receptors on mPFC glutamatergic neurons suggests that their functional interactions may mediate 5-HT effects on this circuit through top-down regulation of amygdala reactivity. Using a multimodal neuroimaging strategy in 39 healthy volunteers, we determined whether threat-related amygdala reactivity, assessed with blood oxygen level-dependent functional magnetic resonance imaging, was significantly predicted by the interaction between mPFC 5-HT_1A _and 5-HT_2A _receptor levels, assessed by positron emission tomography.

**Results:**

5-HT_1A _binding in the mPFC significantly moderated an inverse correlation between mPFC 5-HT_2A _binding and threat-related amygdala reactivity. Specifically, mPFC 5-HT_2A _binding was significantly inversely correlated with amygdala reactivity only when mPFC 5-HT_1A _binding was relatively low.

**Conclusions:**

Our findings provide evidence that 5-HT_1A _and 5-HT_2A _receptors interact to shape serotonergic modulation of a functional circuit between the amygdala and mPFC. The effect of the interaction between mPFC 5-HT_1A _and 5-HT_2A _binding and amygdala reactivity is consistent with the colocalization of these receptors on glutamatergic neurons in the mPFC.

## Background

Research in human and non-human animal models implicates a corticolimbic circuitry composed of structural and functional connections between the amygdala and regions of the medial prefrontal cortex (mPFC) including the anterior cingulate cortex (ACC) in generating and regulating behavioral and physiological responses to threat-related stimuli [1-4]. Regions of the mPFC are crucially involved in the integration and subsequent regulation of stimulus-driven amygdala response, partly via glutamatergic projections to populations of GABAergic neurons within the amygdala [5-7]. Variability in the structure and function of this corticolimbic circuitry has been associated with interindividual differences in personality measures, reflecting sensitivity to environmental threat and related risk for psychopathology [2,8-11].

Serotonin (5-hydroxytryptamine, 5-HT) exerts potent modulatory effects on mood, affect, and responsiveness to stress and threat [12]. Neuroimaging studies in humans have mapped interindividual differences in amygdala reactivity to biologically salient environmental stimuli (for example, facial expressions of threat) onto variability in 5-HT signaling within this corticolimbic circuitry [2,13-21]. However, the role of specific 5-HT-receptor signaling pathways in mediating these effects are not fully understood [12]. Previous work in humans using positron emission tomography (PET) has implicated 5-HT_1A _and 5-HT_2A _receptors in modulating mood, affect and threat responsiveness, and in the corticolimbic circuitry supporting these behaviors [22-26]. Importantly, the anatomical localization of these two receptors within prefrontal cortex positions them to mediate effectively the observed effects of 5-HT signaling on corticolimbic circuit dynamics.

In the mPFC, the excitatory 5-HT_2A _and inhibitory 5-HT_1A _receptors are colocalized on glutamatergic pyramidal neurons [27]. The 5-HT_2A _receptor is specifically localized to proximal portions of apical dendrites [28,29], where convergent inputs are integrated, and is therefore positioned to facilitate mPFC function through second-messenger signaling cascades, resulting in membrane depolarization [27,30]. By contrast, the 5-HT_1A _receptor is localized to the initial segment of the axon, where action potentials are typically generated [27-29,31-34], thus this receptor is positioned to exert an inhibitory effect on mPFC function through 'gating' glutamatergic output via membrane hyperpolarization. Collectively, these two receptors are crucially positioned to mediate effects of 5-HT on glutamatergic neuronal activity and mPFC function, including top-down regulation of amygdala reactivity [27,35].

In one previous study, we identified an inverse correlation between mPFC 5-HT_2A _binding and threat-related amygdala reactivity [14]. In another, we reported that 5-H_1A _autoreceptor binding in the dorsal raphe nucleus was inversely correlated with amygdala reactivity [15]. However, the effects of mPFC 5-HT_1A _binding on threat-related amygdala reactivity were not explored in either of these studies. More importantly, whether mPFC 5-HT_1A _binding moderates the previously observed inverse association between mPFC 5-HT_2A _binding and threat-related amygdala reactivity, as suggested by the aforementioned colocalization of these receptors within the mPFC, has not yet been determined.

In the current study we explored this hypothetical functional interaction using multimodal PET/functional magnetic resonance imaging (fMRI) neuroimaging data in a sample of 39 healthy adult volunteers that partially overlaps with those of our two previous reports [14,15]. We hypothesized that mPFC 5-HT_1A _binding would be positively correlated with threat-related amygdala reactivity, reflecting the inhibitory effects of the 5-HT_1A _receptor on prefrontal pyramidal neurons, which are positioned to exert an inhibitory effect on the amygdala. Consistent with our previous report, we further hypothesized that mPFC 5-HT_2A _binding would be inversely correlated with amygdala reactivity. Finally, in light of the molecular interactions predicted from the colocalization of 5-HT_1A _and 5-HT_2A _receptors, we hypothesized that mPFC 5-HT_1A _binding would significantly interact with mPFC 5-HT_2A _binding, so that 5-HT_2A _binding would be inversely correlated with amygdala reactivity only at relatively low levels of 5-HT_1A _binding.

## Results

### Amygdala reactivity

**Figure 1:**
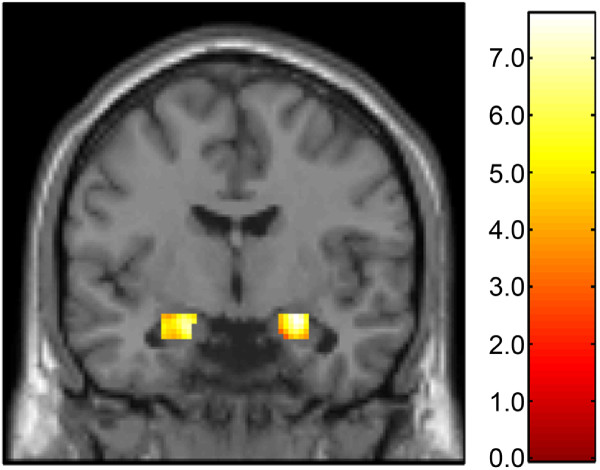
**Amygdala reactivity to perceptual processing of fearful and angry facial expressions**. Statistical parametric map representing bilateral amygdala clusters exhibiting a significant response to task (faces > shapes; right amygdala: (24, -6, -11), z = 6.28, k = 145 voxels (*P *< 0.05, corrected); left amygdala: (-18, -7, -15), z = 5.77, k = 146 voxels (*P *< 0.05, corrected). Color bar indicates t-scores.

Consistent with previous reports, we observed robust threat-related reactivity in the bilateral amygdala across all participants [36,37] (Figure 1). The magnitude of right amygdala reactivity, but not left amygdala reactivity, was inversely correlated with age (right amygdala: r^2 ^= 0.19, *P *= 0.005; left amygdala: r^2 ^= 0.02, *P *= 0.35). Neither right nor left amygdala reactivity was correlated with gender (r^2 ^values < 0.03, *P *values > 0.3).

### Serotonin receptor binding

We focused on quantifying 5-HT_1A _and 5-HT_2A _binding within the pregenual and subgenual prefrontal cortex (pgPFC and sgPFC, respectively) because of previous reports supporting an integral structural and functional relationship between the amygdala and these mPFC regions in the context of processing threat that is modulated by 5-HT signaling [1,2,6,14,38,39].

Reflecting 5-HT_1A _binding, we observed specific [^11^C]WAY100635 binding within both pgPFC (mean ± SD binding potential, non-displaceable (BP_ND_) = 4.32 ± 1.18) and sgPFC (BP_ND _= 4.86 ± 1.41) for all subjects. Reflecting 5-HT_2A _binding, we observed specific [^18^F]altanserin binding within both pgPFC (BP_ND _= 1.06 ± 0.37) and sgPFC (BP_ND _= 1.19 ± 0.46). 5-HT_1A _and 5-HT_2A _binding between the pgPFC and sgPFC were significantly correlated (5-HT_1A _BP_ND_: r^2 ^= 0.69, *P *= 5.87 × 10^-11^; 5-HT_2A _BP_ND_: r^2 ^= 0.63, *P *= 1.80 × 10^-9^). However, within regions, 5-HT_1A _binding was not significantly correlated with 5-HT_2A _binding (pgPFC: r^2 ^= 3.28 × 10^-5^, *P *= 0.97; sgPFC: r^2 ^= 0.001, *P *= 0.88). 5-HT_2A _binding was significantly inversely correlated with age (pgPFC: r^2 ^= 0.41, *P *= 1.30 × 10^-5^; sgPFC: r^2 ^= 0.41, *P *= 1.29 × 10^-5^), but 5-HT_1A _binding did not have a significant correlation with age (pgPFC: r^2 ^= 0.01, *P *= 0.53, sgPFC: r^2 ^= 0.003, *P *= 0.73). Neither 5-HT_1A _nor 5-HT_2A _binding was significantly correlated with gender (r^2 ^values < 0.01, *P *values > 0.5).

### 5-HT_1A _binding and amygdala reactivity

**Figure 2:**
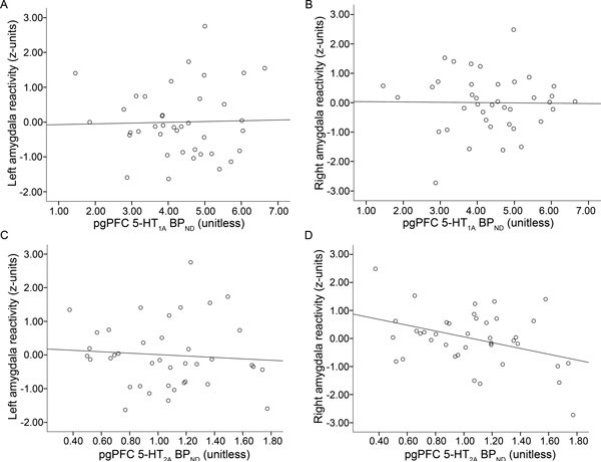
**Association between amygdala reactivity and 5-HT_1A _BP_ND _and 5-HT_2A _BP_ND_**. **(A,B) **Plot of non-significant correlation between left and right amygdala reactivity and pgPFC 5-HT_1A _BP_ND_. **(C) **Plot of non-significant correlation between left amygdala reactivity and pgPFC 5-HT_2A _BP_ND_. **(D) **Plot of significant inverse correlation between right amygdala reactivity and pgPFC 5-HT_2A _BP_ND_. 5-HT = serotonin; BP_ND _= binding potential, non-displaceable; pgPFC = pregenual prefrontal cortex; sgPFC = subgenual prefrontal cortex.

Regional 5-HT_1A _binding was not significantly correlated with amygdala reactivity in either pgPFC nor sgPFC (right amygdala versus pgPFC 5-HT_1A _BP_ND_: t_36 _= -0.76, *P *= 0.94; right amygdala versus sgPFC 5-HT_1A _BP_ND_: t_36 _= 0.54, *P *= 0.60; left amygdala versus pgPFC 5-HT_1A _BP_ND_: t_36 _= 0.16, *P *= 0.88; left amygdala versus sgPFC 5-HT_1A _BP_ND_: t_36 _= -0.09, *P *= 0.93) (Figure 2A,B).

### 5-HT_2A _binding and amygdala reactivity

Regional 5-HT_2A _binding was significantly inversely correlated with amygdala reactivity [14]. Specifically, right amygdala reactivity was inversely correlated with 5-HT_2A _binding within both pgPFC (t_36 _= -3.44, *P *= 0.002; Figure 2D) and sgPFC (t_36 _= -2.49, *P *= 0.02). There was no significant correlation between left amygdala reactivity and 5-HT_2A _binding within either pgPFC (t_36 _= -0.61, *P *= 0.55; Figure 2C) or sgPFC (t_36 _= 0.72, *P *= 0.47). Thus, we focused our analyses on the effects of interaction between 5-HT_1A _and 5-HT_2A _binding on right amygdala reactivity.

### Interaction between 5-HT_1A _and 5-HT_2A _binding and amygdala reactivity

**Figure 3:**
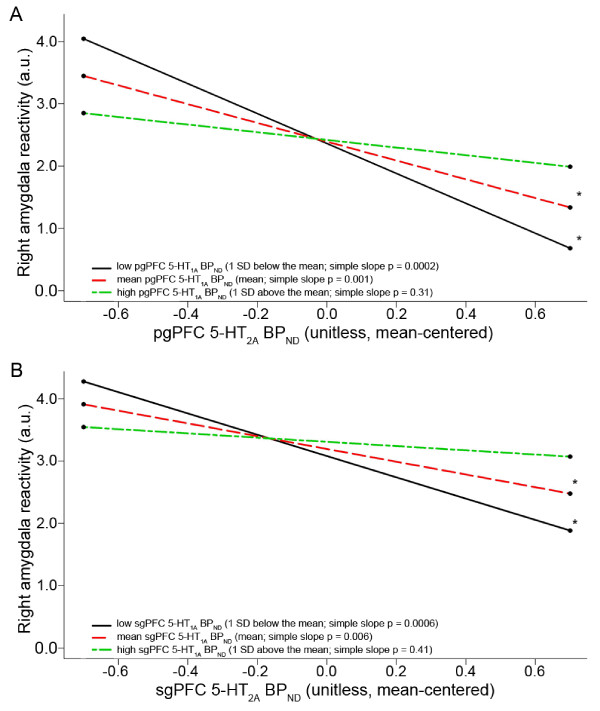
**5-HT_1A _BP_ND _significantly moderated the correlation between 5-HT_2A _BP_ND _and right amygdala reactivity**. **(A) **pgPFC 5-HT_1A _BP_ND _moderated the correlation between pgPFC 5-HT_2A _BP_ND _and right amygdala reactivity. Lines indicate simple slope between pgPFC 5-HT_2A _BP_ND _and right amygdala reactivity at three arbitrarily chosen pgPFC 5-HT_1A _BP_ND _values: low (1 SD below mean (-1 SD), solid black line), mean (equivalent to mean, red dotted line) and high (1 SD above mean (+1 SD), green dotted line). **(B) **sgPFC 5-HT_1A _BP_ND _significantly moderated the association between sgPFC 5-HT_2A _BP_ND _and right amygdala reactivity. Lines indicate simple slope between sgPFC 5-HT_2A _BP_ND _and right amygdala reactivity at three arbitrarily chosen sgPFC 5-HT_1A _BP_ND _values: low (-1 SD, solid black line), mean (red dotted line) and high (+1 SD, green dotted line). *Indicates simple slope, *P *< 0.05; 5-HT = serotonin; a.u. = arbitrary units; BP_ND _= binding potential, non-displaceable; pgPFC = pregenual prefrontal cortex; sgPFC = subgenual prefrontal cortex.

Consistent with our hypothesis, there was a significant interaction between 5-HT_1A _and 5-HT_2A _binding in both the pgPFC (t_34 _= 2.18, *P *= 0.03) and sgPFC (t_34 _= 2.72, *P *= 0.01) in predicting threat-related right amygdala reactivity (Figure 3). Further examination of this interaction effect showed that 5-HT_2A _binding was significantly inversely correlated with right amygdala reactivity when 5-HT_1A _binding was < 4.99 (0.6 SDs above the mean) in the pgPFC or < 5.48 (0.4 SDs above the mean) in the sgPFC. It should be noted that the inverse correlations between 5-HT_2A _binding and right amygdala reactivity remained significant when 5-HT_1A _binding, the interaction term and age were included in the models (pgPFC: t_34 _= -3.55, *P *= 0.001; sgPFC: t_34 _= -2.72, *P *= 0.006).

## Discussion

Results from our current analyses indicate that the interaction between 5-HT_1A _and 5-HT_2A _receptors in the mPFC is crucial for shaping the response of the human amygdala to threat. Specifically, 5-HT_2A _binding was inversely correlated with threat-related amygdala reactivity, but only when 5-HT_1A _binding was at mean or relatively low levels. Importantly, these patterns were independent of age and gender, suggesting the general importance and widespread effects that the interaction between mPFC 5-HT_1A _and 5-HT_2A _receptors may have on amygdala reactivity. The right lateralized nature of this interaction effect may reflect relatively greater involvement of the right amygdala in the perceptual processing of facial stimuli and, subsequently, greater 5-HT modulation of reactivity in this hemisphere. Although a number of studies have reported asymmetries in monoaminergic modulation of cortical and subcortical circuits [40-43], the biological mechanisms mediating such lateralized effects are difficult to ascertain on the basis of the existing literature.

We explicitly tested for an interaction effect (that is, moderation) between 5-HT_1A _and 5-HT_2A _binding because we believe that this represents the most straightforward approach for interpreting how these two systems potentially interact to modulate threat-related amygdala reactivity. Although conceptually and intuitively appealing, we did not employ a metric reflecting the ratio of 5-HT_1A _and 5-HT_2A _binding for two reasons: 1) its association with amygdala reactivity would be arbitrarily dependent upon how the ratio term is constructed and 2) testing for the effect of a ratio term (that is, X_1 _multiplied by the inverse of X_2_) is essentially a test for an interaction effect in which one of the variables is transformed, which we believe would render interpretation difficult at best. Consequently, we believe our test for an interaction between 5-HT_1A _and 5-HT_2A _binding represents the most appropriate and parsimonious statistical test.

**Figure 4:**
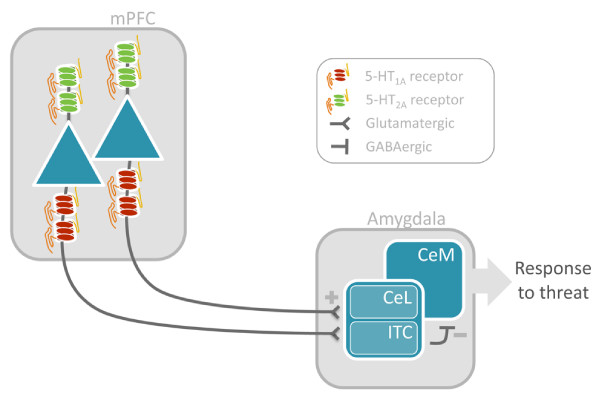
**Schematic illustrating mPFC projection neurons that act to regulate amygdala response to threat-related stimuli**. 5-HT_1A _and 5-HT_2A _in mPFC are positioned to modulate this circuitry by biasing excitability of these mPFC neurons, thereby affecting the capacity to regulate amygdala reactivity. 5-HT = serotonin; mPFC = medial prefrontal cortex; CeL = lateral central nucleus of the amygdala; CeM = medial central nucleus of the amygdala; ITC = intercalated cells.

These findings are remarkably consistent with the predominant anatomical localization of 5-HT_1A _and 5-HT_2A _receptors to the axon hillock and apical dendrites of prefrontal glutamatergic pyramidal neurons, respectively. Given its principal localization on apical dendrites proximal to the soma, the excitatory 5-HT_2A _receptor is situated to mediate 5-HT depolarization of prefrontal glutamatergic neurons. By contrast, the localization of the inhibitory 5-HT_1A _receptor to the initial portion of the axon hillock positions it to mediate 5-HT hyperpolarization of these same neurons. Considering the high coexpression of 5-HT_1A _and 5-HT_2A _receptors on most prefrontal glutamatergic neurons, this arrangement suggests that the 5-HT_1A _receptor can effectively (and negatively) gate the depolarizing effects of the 5-HT_2A _receptors on prefrontal output. In turn, such serotonergic modulation of prefrontal neuron output may shape the capacity of this circuitry to exert an inhibitory effect on amygdala reactivity (Figure 4). We interpret our current findings of an inverse correlation of mPFC 5-HT_2A _binding with amygdala reactivity but only at mean and low levels of 5-HT_1A _binding as reflecting the coexpression of these receptors and their role in mediating serotonergic modulation of this circuitry. The absence of a main effect of mPFC 5-HT_1A _binding on amygdala reactivity is further consistent with this gating model, with the capacity for mPFC 5-HT_1A _receptors to modulate threat-related amygdala reactivity being dependent upon additional signaling mechanisms such as, but not necessarily limited to, mPFC 5-HT_2A _receptors. Although interpretation of our findings is consistent with the previously described localization of the 5-HT_1A _and 5-HT_2A _receptors within prefrontal cortex, our results reflect only statistical correlation, and do not establish causality. Future studies aimed at establishing a causal link between 5-HT_1A _and 5-HT_2A _receptor interactions on prefrontal pyramidal neuron excitability and the response of the amygdala in the context of threat are necessary.

There is strong evidence suggesting that 5-HT signaling within the amygdala plays an important role in modulating threat-related amygdala reactivity [13,20,44], and both 5-HT_1A _and 5-HT_2A _receptors are expressed in the amygdala [45-47]. However, we did not observe a significant correlation between either 5-HT_1A _or 5-HT_2A _binding in the amygdala and amygdala reactivity (data not shown). Unlike in the mPFC, 5-HT_1A _and 5-HT_2A _receptors may be more evenly distributed on both glutamatergic and GABAergic neurons within the amygdala [47,48]. This potential for both receptor subtypes to cause inhibitory and excitatory modulation of the amygdala complicate efforts to map correlations between estimates of local binding and reactivity in the absence of cell-type specific values, which are beyond the scope of current PET techniques. Finally, additional 5-HT receptor signaling mechanisms within the amygdala, such as the 5-HT_3 _and 5-HT_2C _receptors, have been implicated in anxiety-related behavioral phenotypes in animal models, and may have a greater role in mediating the effects of local 5-HT signaling on amygdala function [44,49-51].

There are important limitations to our study. Our blood oxygen level-dependent (BOLD) fMRI challenge paradigm was explicitly designed to elicit threat-related amygdala reactivity associated with driving behavioral and physiologic arousal in response to environmental stimulation. Our task did not engage any mPFC region involved in regulating amygdala reactivity and overlapping with our PET region of interest (ROI). Thus, we were not able to explore the effects of mPFC 5-HT_1A _and 5-HT_2A _binding on mPFC function related to the top-down regulation of amygdala reactivity. Alternative paradigms such as those involving emotion regulation or extinction of conditioned fear responses may help to determine effects of 5-HT_1A _and 5-HT_2A _signaling on related mPFC and amygdala reactivity.

BOLD fMRI and PET receptor imaging provide only indirect metrics of amygdala excitation and 5-HT receptor signaling, respectively. The small sample size in our study limited our power to model interaction effects, thus our findings must be interpreted with caution. The interpretation that our findings reflect the interactive effects of 5-HT_1A _and 5-HT_2A _receptors on glutamatergic neurons is based on evidence that 1) each of these receptors is predominantly localized to glutamatergic neurons [28,32], 2) colocalization of 5-HT_1A _and 5-HT_2A _receptors within the mPFC is predominantly observed on glutamatergic neurons [27], and 3) projections from the mPFC to the amygdala are composed of glutamatergic neurons [52,53]. Despite this, the PET technique does not allow identification of binding associated only with neurons that directly innervate the amygdala, thus we could not confirm the causality of this association using methods currently available. Future studies examining these associations in the context of pharmacological challenge (that is, receptor-specific antagonism) could provide more direct evidence implicating the interaction between mPFC 5-HT_1A _and 5-HT_2A _receptors in mediating the effects of 5-HT signaling on threat-related amygdala reactivity.

## Conclusions

Our current findings provide unique *in vivo *evidence that 5-HT receptors in the mPFC play an important role in shaping interindividual variability in threat-related amygdala reactivity. Specifically, the data reveal that mPFC 5-HT_1A _receptors effectively gate the capacity for mPFC 5-HT_2A _receptors to drive prefrontal pyramidal neuron excitability related to the regulation of threat-related amygdala reactivity. The current work further highlights the effectiveness of multimodal neuroimaging in identifying molecular signaling pathways that modulate neurobiological circuits in humans, and specifically implicates the interaction between mPFC 5-HT_1A _and 5-HT_2A _receptors in modulating the response of the human amygdala and possibly mediating the effects of altered 5-HT signaling on mood, affect and related psychopathology.

## Methods

The study was approved by the institutional review board of the University of Pittsburgh, and written informed consent was obtained from all participants.

### Participants

In total, 39 healthy adult volunteers participated in the study (20 men, 19 women, mean ± SD age 39.1 ± 12.7 years). Subjects were recruited through local advertisements, referrals and ongoing studies. Subjects were generally healthy. Exclusion criteria included 1) current or lifetime mood, anxiety and psychotic disorder as assessed by the Structured Clinical Interview of the fourth edition of the *Diagnostic and Statistical Manual *(DSM-I) [54], 2) family psychiatric history, 3) history of substance abuse or use of antidepressants, 4) early dementia or mild cognitive impairment according to the Mini Mental State Examination [55], 5) reversed sleep-wake cycle, 6) positive test of urine sample for drugs of abuse assessed on the day of scanning. The association between mPFC 5-HT_2A _binding and amygdala reactivity has been described previously involving a subset of this cohort (35 people) [14]. Most subjects completed the fMRI and two PET scan sessions on the same day (n = 33). Those subjects who did not complete all three scan sessions on the same day (n = 6) completed them within 1 month.

### fMRI

#### Protocol

The experimental fMRI paradigm consisted of four blocks of a face-processing task interleaved with five blocks of a sensorimotor control task [14,15]. During the face-processing task, subjects viewed a trio of faces (expressing either anger or fear) and selected one of two faces (bottom) identical to a target face (top). Angry and fearful facial expressions can represent honest indicators of ecologically valid threat, especially that related to conspecific challengers [56]. Based on this, we interpreted the amygdala activation elicited by our task as being threat-related. Subject performance (accuracy and reaction time) was monitored during all scans.

Each sensorimotor control block consisted of six different shapes (circles and vertical and horizontal ellipses) trios. Subjects viewed a shapes trio and selected one of two shapes (bottom) identical to a target shape (top). Each of the six shape trios was presented for 4 seconds with a fixed interstimulus interval (ISI) of 2 seconds, giving a total block length of 36 seconds. Each face-processing block consisted of six face trios, balanced for gender and representing one target affect (angry or fearful) derived from a standard set of pictures of facial affect [57]. Each of the six face trios was presented for 4 seconds with a variable ISI of 2-6 seconds (mean ISI = 4 seconds) for a total block length of 48 seconds. All blocks were preceded by a brief instruction (''Match faces'' or ''Match shapes'') lasting 2 seconds. Total protocol time was 390 seconds.

As we were not interested in neural networks associated with face-specific processing *per se*, but rather in eliciting a maximal amygdala response across all subjects, we chose not to use neutral faces as control stimuli because neutral faces can be subjectively experienced as affectively laden or ambiguous, and thus engage the amygdala [58,59].

#### Acquisition parameters

The acquisition parameters have been described previously [14,15,60]. Briefly, each subject was scanned using a head-only scanner (GE Signa 1.5-T; GE Medical Systems, Milwaukee, WI, USA). BOLD functional images were acquired using a reverse spiral sequence covering 28 slices, each 3.8 mm thick, encompassing the entire cerebrum and most of the cerebellum (repetition time (TR) = 2000 ms, echo time (TE) = 35 ms, field of view (FOV) = 240 mm, matrix = 64 × 64, 195 whole-brain volumes acquired). The first two functional volumes acquired were discarded to allow the scanner to reach equilibrium. Scanning parameters were selected to optimize BOLD signal while maintaining enough slices to acquire whole-brain data. Before the acquisition of fMRI data for each subject, localizer scans were acquired and visually inspected for artifacts such as ghosting, and to ensure good signal across the entire volume of acquisition. Before the acquisition of BOLD data, an auto-shimming procedure was conducted in each subject to minimize field inhomogeneities. The fMRI data for all 39 subjects included in this study were cleared of any related problems.

#### Data analysis

Whole-brain image analysis was completed using the general linear model (GLM) of SPM8 http://www.fil.ion.ucl.ac.uk/spm. Images for each subject were realigned to the first volume in the time series to correct for head motion, spatially normalized into a standard stereotactic space (Montreal Neurological Institute template) using a 12-parameter affine model (final resolution of functional images = 2 mm isotropic voxels), and smoothed to minimize noise and residual difference in gyral anatomy with a Gaussian filter, set at 6-mm full-width at half-maximum. Voxel-wise signal intensities were ratio-normalized to the whole-brain global mean. Preprocessed data sets were analyzed using second-level random-effects models that account for both scan-to-scan and participant-to-participant variability to determine task-specific regional responses.

Variability in single-subject whole-brain functional volumes was determined using the software program Artifact Recognition Toolbox http://www.nitrc.org/projects/artifact_detect. Individual whole-brain BOLD fMRI volumes meeting at least one of the following two criteria were excluded from determination of task-specific effects: 1) significant mean volume signal intensity variation (that is, within-volume mean signal greater or less 4 SDs of mean signal of all volumes in time series); and 2) individual volumes with scan-to-scan movement exceeding 2 mm translation or two degrees of rotation in any direction. On average, 2.1 volumes per subject were excluded because of significant variation in mean volume signal intensity (range of volumes excluded per subject = 0-17), and across all subjects, no volumes were excluded because of excessive motion. Only 1% of all volumes were excluded, thus we believe that this approach enhanced our capacity to determine task-specific effects by excluding volumes with substantial variability without compromising our power to detect task-specific effects by excluding a large number of volumes. We believe this method effectively balances the use of available functional neuroimaging data with a reasonable approach towards accounting for effects due to artifacts or movement.

After preprocessing, our GLM, employing canonical hemodynamic response functions, was used to estimate condition-specific and task-specific BOLD activation for each individual (β weights and contrast images, respectively). Individual contrast images (that is, the weighted sum of the β images) were used in second-level random-effects models to determine mean task-specific amygdala reactivity using one-sample *t*-tests. Group-level effects for our contrast of interest (that is, faces > shapes) were assessed within the amygdala using an ROI constructed from the WFU Pickatlas (version 1.04) [61,62].

To address the issue of multiple voxel-level comparisons, AlphaSim, a software program within AFNI http://afni.nimh.nih/gov/afni that uses a Monte Carlo simulation method, was used to determine that a voxel-wise statistical threshold of *P *< 0.05, uncorrected, combined with a cluster extent threshold of *k *> 56 voxels within our amygdala search volume was sufficiently unlikely (α < 0.05) to have occurred by chance [63]. This threshold was used to assess our main effect of task within the amygdala. Single-subject amygdala-reactivity values for our contrast of interest were extracted from SPM8 using Marsbar (version 0.42) [64]. A sphere of 5 mm radius was centered on the voxel exhibiting the maximal response to our task across all subjects within both the right and left amygdala. Regional 5-HT receptor binding and other variables were regressed against these extracted BOLD values. Neuroimaging data are reported using the coordinate system of Talairach and Tournoux.

### General PET methods

Details concerning the MR and PET imaging procedures related to both [^11^C]WAY100635 and [^18^F]altanserin are described below, and have also been described previously [14,15,65-67] (see previous reports for discussion about the limitations, challenges and methodological attempts to minimize potential artifacts and biases related to these radioligands [25,67-71]).

Structural MR images (GE Signa 1.5-T scanner) were acquired for each subject using a spoiled-gradient (SPGR) recalled sequence (TR = 25 ms, TE = 5 ms, FOV = 240 mm, slice thickness = 1.5 mm, matrix = 256 × 192) with parameters optimized for contrast between gray matter, white matter and cerebrospinal fluid (CSF).

Catheters were placed in an antecubital vein for radioligand injection and in a radial artery for arterial blood sampling. PET scans were acquired using a PET scanner (ECAT HR+; CTI PET systems, Knoxville, TN) in 3D imaging mode (63 transaxial planes, 2.4 mm thickness, 152 mm FOV). Head movement was minimized by use of a thermoplastic mask immobilization system. A 10 minute transmission scan (rotating ^68^Ge/^68^Ga rods) was acquired for attenuation correction of emission data. PET data were further corrected for dead time and scatter.

Each radioligand was administered as a slow bolus over 20 seconds. PET data acquisition and arterial blood sampling was initiated at the start of radioligand injection. The total radioactivity concentration in plasma was determined from approximately 35 0.5-ml hand-drawn blood samples collected over the scanning interval. Additional blood samples were acquired at five to six timepoints during the scan duration for determination of the fraction of the total radioactivity resulting from radiolabeled metabolites of the parent radioligand. Total plasma radioactivity concentration was corrected for radiolabeled metabolites and this 'metabolite-corrected' arterial input function was used for data analysis [69,71].

**Figure 5:**
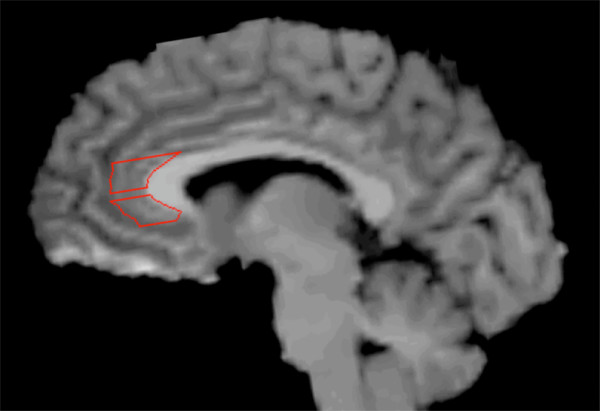
**Sagittal image of single-subject magnetic resonance image with pgPFC (top) and sgPFC (bottom) ROIs outlined**. Despite its appearance, pgPFC and sgPFC ROIs are drawn on consecutive transaxial slices. pgPFC = pregenual prefrontal cortex; ROIs = regions of interest; sgPFC = subgenual prefrontal cortex.

Image reconstruction was performed using filtered back-projection for a final image resolution of about 6 mm. ROIs were drawn on resliced MR images for each subject, and applied to their respective, co-registered PET images (ROIs drawn by SZ and CB). Bilateral ROIs were identified for the sgPFC, pgPFC, amygdala and cerebellum (Figure 5). The cerebellum was used as the reference region for non-displaceable radiotracer uptake (that is, free and nonspecific concentrations, V_ND_) for both [^11^C]WAY 100635 and [^18^F]altanserin.

PET data for both radioligands were analyzed using the Logan graphical method [72] to obtain regional volume of distribution values (V_T_). Regional V_T _values were used to determine the non-displaceable binding potential, BP_ND_, a measure of specific binding. The BP_ND _is directly proportional to B_avail_/K_d_, where B_avail _is the concentration of receptors available for radiotracer binding (that is, not occupied by endogenous 5-HT), and K_d _is the equilibrium dissociation rate constant (that is, inversely related to binding affinity). The PET binding measures were corrected for partial volume effects that arise from atrophy-related CSF dilution using a previously validated two-component MR-based atrophy correction algorithm [66,73,74].

### [^18^F]Altanserin specific methods

The radiosynthesis of [^18^F]altanserin was performed using a modification of the original method [75] that has been used in several studies in our laboratory [14,67,76-78]. [^18^F]Altanserin was administered via intravenous injection (7.23 ± 0.31 mCi), and PET scanning was performed over 90 minutes. The Logan analysis regression was performed over the 12-90 minute post-injection integration intervals (10 points) to obtain regional [^18^F]altanserin V_T _and BP_ND _values.

### [^11^C]WAY100635 specific methods

The radiosynthesis of [^11^C]WAY 100635 was performed as previously described [79], and has been used in several previous studies in our laboratory [15,65,71]. [^11^C]WAY100635 was administered via intravenous injection (14.01 ± 2.10 mCi), and PET scanning was performed over 90 minutes. The Logan analysis regressions were performed over the 14-90 minute post-injection integration interval (13 points) to obtain regional [^11^C]WAY100635 V_T _and BP_ND _values.

### Regression analyses

The association between threat-related amygdala reactivity and 5-HT_1A _and 5-HT_2A _binding was determined using a linear regression analysis between extracted single-subject amygdala BOLD values and ROI-specific 5-HT_1A _or 5-HT_2A _binding values in SPSS (version 17.0; SPSS Inc., Chicago, IL, USA). We previously reported within a subset of this cohort that both amygdala reactivity and mPFC 5-HT_2A _binding are inversely correlated with age [14], and this is consistent with other previous studies [76,78,80]. To account for age-related variability in these two measures, age was included as a covariate in all analyses. Consequently, plots indicate the amygdala reactivity values standardized for age effects. These values are the standardized residuals of amygdala reactivity after accounting for effects of age. This procedure was adopted to illustrate more clearly the relationship between regional 5-HT receptor binding and amygdala reactivity, independent of age. The statistics reported reflect the regression analysis results between observed BOLD and binding values including age as a covariate. As gender was not significantly correlated with our neuroimaging data, it was not included in any analyses determining the relationship between prefrontal 5-HT_1A _or 5-HT_2A _binding and amygdala reactivity.

The association of the interaction between mPFC 5-HT_1A_, 5-HT_2A _binding and threat-related amygdala reactivity was determined using SPSS software and a linear regression model including 5-HT_1A _binding, 5-HT_2A _binding, age and the interaction term as covariates. Additional statistics related to the interaction effects were calculated using a previously validated approach http://www.people.ku.edu/~preacher/interact/mlr2.htm that incorporates parameters estimated from our statistical model (for example, regression coefficients, coefficient covariances) [81]. These additional statistics included simple slopes at specified 5-HT_1A _binding values, significance of simple slopes, and range of 5-HT_1A _binding values over which association between 5-HT_2A _binding and amygdala reactivity was significant.

## Competing interests

The authors declare that they have no competing interests.

## Authors' contributions

PMF designed the study, and participated in data collection, analysis and interpretation and drafting of manuscript. JCP participated in data analysis, interpretation and drafting of manuscript. CCM acquired related funding and participated in interpretation of data. ELMK acquired related funding and participated in interpretation of data. CB participated in data analysis. SLB acquired related funding. ARH designed the study, acquired related funding, and participated in data analysis, interpretation and drafting of manuscript. All authors provided comments and suggestions during manuscript preparation. All authors read and approved the final manuscript.
